# “The Whole Person. Towards a Naturalism of Minds and Persons”

**DOI:** 10.17505/jpor.2025.28096

**Published:** 2025-06-28

**Authors:** Lars-Gunnar Lundh

**Affiliations:** Department of Psychology, Lund University, Sweden

**Keywords:** Bickhard, Naturalism, Autonomy, Mental representation, Philosophy of mind, Concept of person, Sentience

Mark Bickhard is professor at the Department of Philosophy at Lehigh University in Bethlehem, Pennsylvania, US. Although primarily a philosopher, one of his main interests is theoretical psychology. In this new book, he attempts to lay the foundations for a naturalistic understanding of minds and persons. His big question is how we can arrive at an integrated understanding of the world, including minds and persons. As he puts it in the beginning of the book:

Can we understand human beings—minds and persons—as a natural part of that world? Naturalism has succeeded in integrating almost all of the world… The one remaining holdout against this success of naturalism is: persons. Mind and, more broadly, *persons* still resist naturalistic understanding. There are even those who contend, or perhaps hope, that persons will remain exceptions to naturalism. They maintain that persons are fundamentally different in kind from anything else, and that naturalistic understanding of persons, therefore, is simply impossible…

The central thesis of this book is that such a naturalism is not only possible, but that its outlines are becoming clear. The ultimate goal is to move forward toward a naturalism of the whole person—one of the last chapters that remains to be written in the historical drama of naturalism. (Bickhard, 2025, p. 3)

Bickhard’s book contains nearly 500 pages and what can be done in a brief review is merely to provide some illustrations of his approach to the big and complex questions that are involved. I have chosen to focus on three concepts that are central to this discussion: naturalism, autonomy and mental representation. I will start with a brief discussion of the concept of “naturalism” and of how Bickhard wants to make “free will” and personal responsibility (which are commonly attributed to persons) compatible with a naturalistic understanding of human beings. Second, I will turn to Bickhard’s way of tracing the development of autonomy from unicellular organisms to human beings. And third, I will discuss his interactivist approach to the understanding of mental representations. Finally, I will turn to what I perceive as one of the limitations of Bickhard’s work – his relative silence about another central aspect of persons and minds: sentience.

## What is a Naturalistic Understanding of Persons?

As stated in the Stanford Encyclopedia of Philosophy, *naturalism* means to believe

that reality is exhausted by nature, containing nothing “super-natural”, and that the scientific method should be used to investigate all areas of reality, including the “human spirit”. (Papineau, 2023)

Although this may seem to be a rather uncontroversial claim, there are several complications involved. First, as Bickhard points out, naturalism “can take multiple forms depending on how the natural world is itself construed”. For example, if the natural world is assumed to consist of “substances” or “particles” that are related by means of causal chains, the functioning of minds and persons may easily be set aside as “non-natural” (Bickhard, 2025, p. 13).

In other words, there are certain *forms* of naturalism that make it difficult to see minds and persons as part of nature. Bickhard, however, firmly rejects the notion of the natural world as consisting of “substances or particles together with their factual and causal relations”. Instead, he argues for a perspective where the natural world is conceptualized in terms of *processes* (i.e., a “process metaphysics”, rather than a “substance metaphysics”). Moreover, he argues that the development of the natural sciences involves a successive *replacement of substance models with process models*:

Thus, many of the successes of the naturalistic approach have involved the replacement of substance conceptions of some phenomena with process conceptions. They have involved a replacement of a substance metaphysics with a process metaphysics, at least with respect to the phenomena at issue. Fire is no longer modeled as the release of the substance phlogiston, but as the process of combustion; heat is no longer conceptualized in terms of the substance caloric, but rather as a process of random kinetic motion; life is no longer held to inhere in a substance of vital fluid, but rather as a special kind of self-reproducing far from thermodynamic-equilibrium process. The history of naturalism, the history of science, is a history of the replacement of substance models with process models (Bickhard, 2025, p. 15-16).

Bickhard’s naturalism, in other words, differs from some other varieties of naturalism by its conceptualization of nature in terms of *processes*, rather than substances.

Bickhard argues convincingly that the reason why many philosophers and psychologists have had difficulties with the concepts of mind and person is that they operate within models where the natural world is conceptualized in terms of substances or particles, rather than processes. The critical point, as he states it. is that processes are inherently *organized*. Processes exist at many levels of complexity, and at the biological and psychological levels we find complex processes of emergent *self-organization*. These processes cannot be reduced to causal chains:

the world is not constituted out of causal chains. There are also complex dynamics, including self-organizing dynamics, that cannot be rendered in terms of collections of causal chains (Bickhard, 2025, p. 390).

Consider, for example, Ossorio’s (2006) definition of the person as an individual with a life history of patterns of *deliberate action*. Persons are seen as agents who can *deliberate* about their alternatives of action before they act, and who can then *choose* what to do based on their deliberations. This seems to implicate some form of “free will”. And how can such a notion possibly be compatible with a naturalistic understanding of the world?

Bickhard is very clear that people make deliberate choices about how to behave, and that they are responsible for their actions – otherwise they would not be persons – but their decisions are in turn the product of larger processes:

if a person decides with full consideration of alternatives and values to engage in morally questionable acts, but someone claims that they are nevertheless not responsible because everything they do is determined, then this simply overlooks the fact that — determined or not — those acts depended essentially on that particular organization of considerations and values and hierarchical prioritizations of those values, etc. that constitute that person. Those acts do depend on the person, however much it may also be the case that that person is in turn (causally) dependent on prior and external processes. (Bickhard, 2025, p. 389)

In other words, there is a kind of complementarity between understanding a person’s actions in terms of their *inner dynamics* and analyzing these actions in terms of the *historical origins* of these dynamics.

There is a similarity here to the argument about etiological models of function: it is the current dynamics that are crucial, not the origins of (a system with) those dynamics. Such historical considerations are important — for example, for understanding those origins, and, consequently, for the policy kinds of considerations mentioned—but they do not affect the current dynamic or action possibilities of the organism. (Bickhard, 2025, p. 390*n*)

Free will is sometimes spoken of as if it were “free” in some absolute sense – that is, as if acts of will were some kind of “first causes” in a causal chain. Bickhard rejects such claims by rejecting the model of *causal chains* which, as he points out, “vitiates much of action theory, as well as the narrower framework concerning issues of free will” (p. 390). As he puts it,

Among other multiple problems involved in trying to model actions as causal chains is that it is conceptually rather difficult to figure out how to initiate a causal chain de novo. How do you initiate the initiation of your action? How do you decide to decide (to act)? In terms of what reasons do you reason? This family of problems has no solution within a causal chain framework other than stipulative fiat about some kind of “act” being the first one that initiates the chain; otherwise there ensues a regress to attempt to close off the bare, uncaused, first event of the causal chain. The problems disappear if emergent self-organization is taken as a model for decision-making and interaction initiation and guidance, but that requires giving up causal chains constituted out of metaphysical events. (p. 391)

In Bickhard’s view, the person is seen as “a complex organization of processes” (p. 410), and persons are said to “self-organize processes that ongoingly monitor and control interaction processes” (p. 390).

Importantly, this capacity for self-organization is *not* something that appears for the first time in human beings. On the contrary, the development of self-organizing living systems can be traced back through evolution in what could partly be described as “the natural history of autonomy” (although this is not a phrase used by Bickhard).

## A Natural History of Autonomy

Bickhard describes a conceptual framework that relies on dynamic system theory and emergentism. Emergentism is based on the assumption that the world is constituted by *processes*, rather than by particles, entities or substances, and that this makes *organization* into a causal factor. Importantly, processes are inherently organized, and differing organizations can yield different causal influences on the world.

Processes vary in their degree of stability. Some processes (e.g., a rock falling) are not stable and go to completion rather quickly. Other processes are much more organized and stable over time. Of particular interest is the subclass of relatively stable processes that Bickhard describes as processes that are *intrinsically far from thermodynamic equilibrium* and that cease to exist when they go to equilibrium. A simple example is a candle flame, which is maintained only as long as its temperature is kept above the combustion threshold. The crucial point is that such systems are stable only if they are maintained in their “far from thermodynamic equilibrium conditions”. A candle flame can maintain itself by making use of its environment to maintain a temperature above the combustion threshold, by processes such as vaporizing wax, inducing convection to bring in fresh oxygen and the disposing of waste products. It is thereby an example of a self-maintaining process, although severely limited in this capacity as compared with living organisms.

If a candle is running out of wax, there is no alternative process available to the candle. In contrast, living systems have such alternative processes available; they can *switch* between *alternative* ways to remain self-maintaining by *changing their interaction with the environment*. More specifically, Bickhard argues that living systems are characterized by *recursive* self-maintenance.

The concept of *recursive self-maintenance* is central to Bickhard’s view – living systems maintain their property of being self-maintaining by shifting between different processes of interacting with the environment. This represents a form of *autonomy* and is an ability that comes in degrees. Even unicellular organisms such as bacteria show simple forms of such switching (e.g., between swimming and tumbling) as part of their self-maintenance:

Swimming is a contribution to the self-maintenance of the bacterium if the bacterium is pointed up a sugar gradient; it is deleterious if it is pointed down a sugar gradient. Conversely, tumbling contributes to self-maintenance if it is pointed down a sugar gradient, but not if it is pointed up the gradient. The bacterium can select between swimming and tumbling in ways that are (generally) appropriate to its conditions in order to do what will be self-maintaining in those conditions. It will tend to maintain its condition of being self-maintenant, thus recursive self-maintenance.

In order to engage in such switching, the bacterium must have some means of detecting, however fallibly, its orientation relative to sugar gradients, means by which it can swim and tumble, and a way to switch between swimming and tumbling appropriately on the basis of the orientation detections. Such processes must remain stable relative to the activities of swimming or tumbling per se — they must involve infrastructure. (Bickhard, 2025, p. 111)

This may be an illustration of the simplest possible form of recursive self-maintenance and autonomy. In bacteria, the switching is accomplished by a relatively simple *triggering* mechanism. More complex forms are seen in multicellular organisms with nervous systems:

A frog, for example, might have the option of flicking its tongue one way to eat a fly or another way to eat a worm. These must both be *indicated*, and some sort of selection made between them. Such an indicative relationship replaces the triggering connections, and the further selection process replaces the triggering process as initiator of interaction (Bickhard, 2025, p. 111)

The bacterium can hardly be said to “choose” between swimming and tumbling – here the response is triggered by the momentary stimulus conditions. But in the example with the frog, there appear two alternative possibilities of action at the same time, and a selection between these alternatives is made. This, of course, requires that these possibilities, as Bickhard expresses it, are *indicated* to the frog in a way that makes it able to perceive these possibilities. At the same time, this also illustrates how the frog shows a higher degree of autonomy than the bacterium.

The development of autonomy in living beings has been a theme also in the writings of other theorists such as Varela et al. (1991) and Thompson (2007). In their writings, however, there is more emphasis on the *independence* of the system from its environment (e.g., on the reproduction of internal components and processes), whereas Bickhard emphasizes the *interrelations* to the environment – that is, the ability to make use of the environment’s resources for the sake of self-maintenance.

Importantly, what is at stake here seems to be the capacity of living organisms to relate to alternative *possibilities* of interacting with the environment. This is clearly consistent with Gibson’s (1966, 1979) theory of *affordances*. In Gibson’s (1979) ecological approach to perception the most important information that is provided via our senses is information about the environment’s affordances, defined as what the environment offers the individual in terms of possible interactions, for good or bad.

One difference between Bickhard and Gibson, however, is that whereas Gibson rejects the need for any concept of mental *representation*, Bickhard develops an interactivist model of representation. Both Bickhard and Gibson raise similar arguments against traditional models of mental representations, that lead to infinite regresses and homunculus-like notions. Whereas Gibson entirely rejects the concept of mental representation, Bickhard argues for a notion of representation as a *process*:

we do not have or perceive mental representations; we engage in (mental) processes or activities of (that constitute) representing. (Bickhard 2025, p. 195)

## The Nature of Mental Representation

As I understand Bickhard’s reasoning, mental representation *emerges* with the evolution of living beings who can relate to alternative *possibilities* of interacting with the environment – as distinct from just interacting with the actual situation. Relating to possibilities means to *represent* these possibilities. This commonly takes the form of *anticipations* about what will take place *if* the individual acts in this or that way in a certain surrounding. In Bickhard’s terminology, this means that these anticipations involve (i.e., contain) *functional presuppositions* about the nature of the environment that is being interacted with. Since such functional presuppositions (“beliefs”) need not always be correct, it also means that the living being can detect mistakes and make corrections if the anticipated outcome is not realized. In other words, truth and falsehood now appear on the scene, first in the form of practical *success or failure*, and then (at least in human beings) in the possibility of detecting *errors* in one’s own inner representations (“functional presuppositions”) by means of reflection.

An interesting thing about these interaction possibilities is that they can form *complex webs*, as Bickhard puts it. That is, in relatively complex organisms, there is not only selection between two possible ways of interacting with the environment. Depending on which alternative is chosen, *whole new horizons of possibilities* open up to the organism (Bickhard doesn’t use the term “horizon”, but to me it seems to be an apt term).

the potentialities for indicating interaction possibilities can be linked and iterated: if one interaction were to be engaged in and completed, that could serve to indicate further possibilities of interaction, each of which could, in turn, indicate still others. If the frog flicks its tongue in a particular way, then eating may become possible; if the frog moves slightly to the left, new tongue-flicking and eating possibilities may come in range. Indications of interactive potentialities, thus, can branch with multiple possibilities and can iterate in conditionalized indicative relationships. Indications of interactive potentialities, then, can form webs, perhaps complex webs, of interaction potentialities conditional on other interaction potentialities being engaged in and completed in indicated ways. (Bickhard, 2025, p. 111-112)

All this points in the direction of the development of the exceedingly complex forms of deliberate action, and autonomy, that characterize human beings, and which are typically seen as essential to the notion of personhood. The beauty of Bickhard’s reasoning is that it shows how we may find a whole spectrum of such processes at different levels of complexity also in other species, ranging from simple forms of switching between alternative behaviours to the selection of complex behaviours based on successively more complex webs of interaction potentialities.

What then is the nature of these processes of representation? Bickhard makes clear that his model is a *three*-parts model, which contains *representation, content*, and *represented*. He compares this model with *two* parts-models (that contain only representation and represented) and with other three-parts models. The relation between representation and content in Bickhard’s model is a logical, *internal* relation – more specifically, the functional presuppositions (the content) inhere *implicitly* in the anticipations (the representation) and can be made explicit by means of conscious reflection. The relation to the *represented*, on the other hand, is an empirical, *external* relation, which is open to falsification and correction by interactions with the environment.

According to Bickhard, this three-parts model solves two basic problems with other models of representation: (1) It can account for the possibility of error (via the *external* relation to the represented), and (2) it does not (because of the *internal* relation between representation and content) require the postulation of any inner “homunculus” to interpret the representation.

If a representation is related to its content externally, then, by that assumption, there is no inherent connection between that representing element and its content… The assumption that content is carried in an external relation forces that that content be provided by some sort of homunculus. When such a homunculus can be easily accounted for, such as for the user of Morse code or the user of a computer program, that is not a problem. But when mental representation is what we are attempting to understand, then such a requirement for a homunculus simply renders the account circular or initiates a regress.

The only way in which to halt such a regress is with some form of representation for which the content is carried *internally, intrinsically*. If something carries a representational content in virtue of the nature of what that something is, then there is no need for an interpreter either to provide the content in the first place or to interpret the representation as having that content when the representation is used.

Indications of potential interactions carry their functional presuppositions internally. They could not be indications of those interactions as being appropriate within and for this particular recursively self-maintenant system without having those presupposed conditions of success, of appropriateness. No homunculus is required for those presuppositions to be presupposed. The functional presuppositional relationship is an internal relationship. (Bickhard, 2025, p. 116)

The contrast with Morse code is illuminating. Morse code works on a two-parts *encoding* model of the correspondence between representation and represented. In Morse code, a sound signal *encodes* a letter, which is useful because the signal can be sent over telegraph wires while the letter cannot. But it requires an external user and interpreter to function. With mental representations it is otherwise, since there is no internal interpreter inside a person’s head (a “homunculus”) that can interpret the representation. Mental representations therefore must be understood on the basis of some other model than the *encoding* model.

Yet, a basic problem with much theorizing in psychology is precisely that it has tried to use encoding models to understand mental representations. Bickhard refers to this as *encodingism* and argues persuasively and in a great detail why this way of thinking does not work if we are to understand the nature of mental representations. Mental representations are completely different from man-made codes such as Morse code and computer programs. Mental representations *are not encodings*.

When we are interacting with the environment, according to Bickhard’s model, our experience is characterized by anticipations of what will happen in the near future. These anticipations contain functional presuppositions about the nature of the environment we are interacting with, and about what will take place if we do this or that. The anticipations are *explicit*, whereas the functional presuppositions are *implicit*. The functional presuppositions may be correct or not, and if they are incorrect this may lead to cognitive change.

Bickhard describes the implicit functional presuppositions as a “readiness” in the individual to anticipate certain consequences *if* they act in a certain way in a certain kind of situation. This means that the representations are intrinsically related not only to motivation (i.e., what is valued by the organism) but also to *differentiation*. Here again, Bickhard’s thinking seems to converge with Gibson’s approach to perception, according to which perceptual learning is seen as differentiation (Gibson & Gibson, 1955). A main difference, however, is that Bickhard does not stay at the level of perception. For example, he points to the role of differentiation for the implicit definition of the *categories* into which an animal structures its world. He also emphasizes the role of perception in the development of the individual’s *situation knowledge*, and the more encompassing *world knowledge*, and the continual updating of this knowledge by means of ongoing interactions with the environment – a process that he refers to as *apperception*.

An additional development in humans is their capacity for *reflective* consciousness, as a “second-order knowing” that is based on an interaction with the processes at the “first-level of knowing” – as seen, for example, in the internal rehearsal of thoughts, and the checking out of various possibilities in imagination without having to engage in explicit interactions with the environment.

There is much to say about Bickhard’s interesting analyses of the complexities in human representing of the world, but I will finish this part of the review by some short notes on his application of the interactivist model to the social realm. Here he points to a fundamental difference between human knowledge of the physical environment and their knowledge of the social world. As to the physical environment, the continuous updating of knowledge, based upon prior interactions and their outcomes, makes it possible to arrive at relatively reliable predictions of where further interactions may lead. As to the social world, however, it becomes much more complicated, because other individuals act on the basis of internal processes that are not open to direct observation. Further, they are

sensitive to *other* agents in their presence, and here an additional complication emerges: If I am to fully characterize the interactive potential of a situation that contains you in my presence, part of that characterization must include a characterization of the interactive potential that you afford. But you are an agent, with hidden internal conditions, which include your own characterization of your environment, which, in turn, includes me — and my characterization of you. So, a complete characterization of my environment must include you and your characterization of me, including your version of my characterization of you, and so on. (Bickhard, 2025, p. 262-263)

As Bickhard describes it, this creates an epistemological problem for the partners in any social interaction – a problem that must be solved if their interaction is to proceed successfully. In other words, they must arrive at a mutual and mutually consistent characterization of the social situation. This is described as a problem of *coordination*, and the solution as a *situation convention*. Bickhard sets his task here

to model what these could possibly be, how they could be established, explore their properties, and explore any further emergents involving them. A first point is that such situation conventions do constitute a level of emergence beyond that of individual agents.

## The *Whole* Person?

Bickhard’s book is extremely rich in content. To provide the reader with at least some idea of these contents, a short list may be given of examples of issues that are discussed in the book. The book contains twelve chapters. The first five chapters, which fill the first 80 pages of the book, are rather brief. After a brief introductory chapter, there follow chapters on naturalism, background metaphysics and epistemology, historical perspectives on thinking about these matters, and a chapter on emergence.

Then comes three chapters on biological foundations. In the first, Bickhard describes dynamic systems terms in terms of self-organizing emergent processes with dynamic landscapes and attractors, niches, modulators of metabolism, primitive forms of autonomy, multicellularity, DNA as regulatory resource, and enabling hierarchies. The second of these chapters is about the emergence of normativity; here he elaborates on the dynamical model of function, compares and contrasts recursive self-maintenance with homeostasis, discusses the emergence of representational normativeness, and presents detailed analyses of his model of representation as compared with alternative models. In the third chapter he describes a ”macro-evolutionary ratchet” of basic human capacities in four steps: the development of interactive knowing, learning, emotions, and reflective consciousness.

After this, there are two long chapters on minds and person, respectively. The first of these chapters, which is entitled *The mentality of Homo Sapiens*, contains almost 100 pages with further elaborations of interactive knowing, discussions of motivation, microgenesis, the central nervous system as a dynamic system, agency, thinking and conceptualization, holism, attention, memory, development, consciousness, intentionality, and much more. The second chapter in this part of the book, which is even longer (more than 180 pages) has the thought-provoking title *Persons: The emergence of Homo Socius*. Here Bickhard discusses the ontological emergence of human sociality, with a focus on social conventions and language, but also on developmental issues concerning language, cognition, rationality, culture, personality, psychopathology, and ethics. Finally, the book is rounded off with two rather short chapters on reflexive consistencies and naturalistic ontological psychology.

In view of these widely encompassing contents, it might be taken for granted that the book really deserves the title *The Whole Person*. But despite my enthusiasm about the book, I cannot help feeling that it is heavily biased towards cognitive science. There *are* sections, for example, on emotion, but these are rather brief and contains nothing like the detailed analysis of different theories that are found in other parts of the book.

There is even less on *feeling*. For example, I would have liked to see a discussion about *sentience* and how it fits into a naturalistic perspective on the development of the human mind and personal development. This is an aspect of consciousness which is not mentioned by Bickhard when he summarizes his view on consciousness as involving

two different though intrinsically related phenomena: (1) a basic flow of awareness involving varying degrees and kinds of normativities, including representing processes and (2) possibilities, in some species (not all), of reflection on and interaction with such processes. (Bickhard, 2025, p. 455)

Sentience has been defined as a capacity for subjective experience, sensations and feelings (e.g., Thompson, 2022). Important examples are the experience of pleasure and pain, thirst and hunger, sensations of taste and smell, and bodily feelings in general.

Bickhard does approach this issue in some passages, where he mentions experiencing and so-called “qualia”, that is, qualitative aspects of experience. Somewhat unexpectedly, however, he seems to reduce experiencing to purely cognitive processes, such as anticipations.

Experiencing is… a flow of anticipating of future potentialities for further process, interactive process in particular. It is a flow of the anticipating of the organization of future potential, with the present as the locus from which that organization proceeds. (Bickhard, 2025, p. 251)

The experience of qualia is not seen as a part of *primary* experiencing but as products of *reflective* consciousness:

Qualities of this flow… are neither explicit nor constitutive. They can become themselves experienced, however, in reflection… Those qualities of experiencing will be implicit in primary experiencing in the sense that they are potentialities that are realized in reflection. But they are not explicit in primary experiencing and they are not constitutive of primary experiencing… Qualia, in a sense, are constructed in and by reflective analysis of primary experiencing (Bickhard, 2025, p. 253)

All this seems to imply that feelings are “implicit” until they are *reflected* upon. Bickhard’s position also seems to imply that only animals who are capable of reflective consciousness have feelings. This is a very restrictive notion of sentience, that seems to be at odds with most other thinking in this area. A rather common hypothesis in the literature on this topic is that sentience is found in all organisms who are capable of learning. However, more radical suggestions have also been made. According to Reber and Baluška’s (2021) Cellular Basis of Consciousness (CBC) model, for example, sentience and life are coterminous. According to their model, all organisms are sentient, that is, have subjective experiences and feelings, based on inherent cellular activities via processes that take place in excitable membranes of their cells.

The questions involved here are complex, and further analysis may probably require more refined distinctions between different aspects of experiencing. Godfrey-Smith (2024), for example, describes an approach called the neural dynamics of subjectivity (NDS), and argues that an evolutionary perspective

motivates a strongly gradualist view of consciousness; a simple distinction between conscious and nonconscious animals will probably be replaced with a view that admits differences of degree, perhaps on many dimensions” (p. 1660).

Importantly, sentience may be basic to *self*-experience. According to Gibson’s (1966) theory of perception, perception is not based on sensations, but on the pick-up of information about the affordances of the environment. Still, Gibson admits the essential importance of sensations for the awareness of the *self*.

Perhaps whatever specifies the organism as existing in its environment is to be called sensation. The temporary array of perspective appearances of the world is called the field of visual sensations, or the visual field, and this, I think, is the best index an observer has of himself as *here*. So I have to admit that the study of sensations is important for an understanding of one’s awareness of the self even when I deny that it is basic to an understanding of one’s awareness of the world. (Gibson, 1969, p. 409)

If sensation, as Gibson suggests, is “whatever specifies the organism as existing in its environment”, it must be seen as representing a very basic aspect of organismic functioning.

From a whole-person perspective on human beings, this has been discussed in terms of *experienced embodiment*, and it has been suggested that the experience of how the body *feels “from within”* (the body I *am*, as distinct from cognitions about the body I *have*) is basic to the development of self-identity and mental health (Lundh & Foster, 2024). Unfortunately, this whole dimension is missing from Bickhard’s book. When he writes of self and identity it is done from a cognitive perspective, with a focus on the “worldly aboutness” of the individual’s experiences, and no focus on how it *feels* to be an embodied individual in that world. In this sense, it might be questioned whether this book is really about the *whole* person. This does not, however, in any way detract from the importance of Bickhard’s work.

## Conclusion

This is an important work, which argues persuasively for how the human mind, and personal development, can be understood from a naturalistic perspective. The perhaps most crucial point is Bickhard’s arguments for *process* models in science – this is probably what will make it possible to integrate psychology with other sciences, without giving up on notions such as free will and personal responsibility. His concept of recursive self-maintenance and his interactive approach to the understanding of representing/representation also belong to the many things that stay with me after reading this book.

The book could perhaps have been better organized. Its 500 pages contain a lot of repetitions, and more editing would probably have made the book more readable. Still, Bickhard’s book is a veritable gold mine for anyone who has theoretical psychology as one of their passions, and I will surely return to it again for a second (and probably also for a third, and fourth) reading.


Lars-Gunnar Lundh


## Open access

This article is distributed under the terms of the Creative Commons Attribution 4.0 International License (http://creativecommons.org/licenses/by/4.0/), which permits unrestricted use, distribution, and reproduction in any medium, provided you give appropriate credit to the original author(s) and the source, provide a link to the Creative Commons license, and indicate if changes were made.
